# Comparisons of postoperative outcomes of laparoscopic versus open surgery using inverse probability of treatment weighting analysis: an evidence from Iran

**DOI:** 10.1186/s12893-024-02389-0

**Published:** 2024-06-14

**Authors:** Zahra Jamali, Mahboobeh Pourahmad, Hajar Khazraei, Faranak Bahrami, Mohsen Bayati, Saeedeh Pourahmad

**Affiliations:** 1 Student Research Committee , Shiraz University of Medical Sciences, Shiraz, Iran; 2 Colorectal Research Center, Shiraz University of Medical Sciences, Shiraz, Iran; 3 Health Human Resources Research Center, School of Health Management and Information Sciences, Shiraz University of Medical Sciences, Shiraz, Iran; 4 Biostatistics Department, Medical School, Shiraz University of Medical Sciences, Shiraz, Iran

**Keywords:** Colorectal cancer, Laparoscopy, Surgery, Laparotomy, Treatment outcome, Propensity score

## Abstract

**Background:**

Colorectal cancer has created a significant burden worldwide, including in Iran. Open and laparoscopic surgery are important treatment methods for this disease. The aim of this study is to compare postoperative outcomes of laparoscopic versus open surgery in Iran, with a particular emphasis on controlling confounding factors.

**Methods:**

To control confounding factors in between-group comparisons of observational studies, a method based on propensity scores was used. The current study was conducted on 916 patients with colorectal cancer in the city of Shiraz between the years 2011 to 2022. The required data regarding treatment outcomes, type of surgery, demographic characteristics, and clinical factors related to cancer was extracted from the Colorectal Cancer Research Center of Shiraz University of Medical Sciences. To control confounding factors, we used the Inverse Probability of Treatment Weighting (IPTW) as one of the analytical approaches based on Propensity Score analysis. After IPTW analysis, univariate logistic regression was used for treatment effect estimation. Stata 17 was used for statistical analysis.

**Results:**

After controlling for 24 clinical and demographic covariates, negative post-operative outcomes were significantly lower in laparoscopic than open surgery. There were significant differences between the two groups of surgery in the percentages of death due to cancer (*P* < 0.01), recurrence (*P* < 0.01), and metastasis (*P* < 0.05). The treatment effect univariate logistic regression analysis indicated that laparoscopic surgery reduced the risk of negative postoperative outcomes including death due to cancer (OR = 0.411, *P* < 0.01), recurrence (OR = 0.343, *P* < 0.01) and metastasis (OR = 0.611, *P* < 0.05) compared to open surgery.

**Conclusions:**

In terms of postoperative outcomes including cancer-related mortality, recurrence, and metastasis, the laparoscopic surgery outperformed open surgery. Therefore, further development of laparoscopic surgery can lead to better health outcomes for the population and optimize the utilization of healthcare resources.

## Background

According to the latest report from the Global Burden of Disease (GBD), more than 26 million new cases of cancer were reported worldwide in 2019, resulting in approximately 10 million cancer-related deaths [1]. In Iran, cancer is also recognized as the second leading cause of death, with approximately 56,000 deaths and over 100,000 new cases reported in 2018, according to the World Health Organization (WHO) [2]. Among cancers, colorectal cancer ranks second in terms of the highest disease burden, with a reported mortality rate of 1,090 per 100,000 individuals. In 2019, over one million people died from colorectal cancer, and there were 2.17 million new cases reported [3]. The prevalence of this cancer has been on the rise in recent years. In Iran, colorectal cancer ranks third among all cancers, with approximately 10,000 new cases occurring annually [2].

Colorectal cancer or carcinoma occurs when the cells that line the colon or rectum become abnormal and grow uncontrollably. Several risk factors for developing this cancer include increasing age, tobacco use, obesity, and lack of physical activity. Symptoms of colorectal cancer may include rectal bleeding, sudden weight loss, abdominal pain and cramping, and anemia for unknown reasons [4].

Surgery, chemotherapy, and radiation therapy are among the treatment options for cancer. In surgery, a portion or all the affected tissue is removed. In chemotherapy and radiation therapy, drugs and radiation are used to destroy cancer cells or slow down their growth. The choice of treatment method depends on the stage and severity of the cancer, but surgery is the most common approach and can be performed through various methods, including laparoscopic surgery and open surgery (laparotomy). In laparoscopic surgery, several small incisions are made on the patient’s abdomen, and the cancerous area is removed. The recovery period and hospital stay for this method are shorter compared to laparotomy. The long-term effectiveness of laparoscopic surgery compared to open surgery is still being studied [5]. For example, a meta-analysis on 12 randomized controlled trials (RCTs) concluded that laparoscopy had better short-term outcomes compared to open surgery, but there were no significant differences in long-term outcomes such as overall mortality, cancer-related mortality, and disease recurrence between the two surgical approaches [6].

Interventional studies are not always feasible due to practical and ethical limitations, as well as cost and time constraints. Therefore, the use of observational studies to examine the effects of interventions becomes necessary [4]. In observational studies, the treatment choice is influenced by the characteristics of the physician, patient, and the healthcare system. One of the challenges of non-randomized observational studies is that if a difference is observed between treatment groups, it cannot be solely attributed to the intervention, and this difference may be influenced by other characteristics or variables, leading to biased estimates of the treatment effect. Therefore, in these studies, it is important to control for these confounders [7]. One of the most well-known methods for controlling confounding in observational studies is propensity score (PS) analysis. The PS is a value that summarizes all measured pre-treatment characteristics. In other words, the PS represents the conditional probability of receiving a specific treatment given the observed covariates, or receiving another treatment or no treatment. Individuals in the treatment groups with equal PS values are, on average, similar in pre-treatment characteristics [8]. One approach within this framework is the Inverse Probability of Treatment Weighting (IPTW), where a weighted artificial population is created. Individuals in the treatment group are assigned weights equal to the inverse of their PS, while individuals in the non-treatment group are assigned weights equal to the inverse of (1 - PS) [7].

Based on the status and postoperative consequences in patients with colorectal cancer, which directly affects the quality of life of patients, and considering the relatively high prevalence of this disease in Iran, comparison of the adverse effects of surgical methods seems necessary. In this regard, the use of statistical methods such as PS analysis, which is performed to control confounders and reduce bias in the comparison between interventions in observational studies, is important. The searches conducted on this subject indicate limited use of PS analysis in Iran, particularly in the case of cancer. Furthermore, most studies conducted worldwide have focused on PS matching, while other PS analysis methods have received less attention. Due to the higher retention of individuals in the IPTW compared to PS matching, this method is of interest. The present research aimed to compare postoperative complications such as the status of survival, metastasis, and recurrence of the disease between surgical groups in treatment of colorectal cancer using inverse probability weighting through PS by logistic regression method to reduce non-random allocation bias and control confounders.

The high prevalence of colorectal cancer in Iran and the lack of a rigorous study to compare the effectiveness of laparoscopic surgery versus open surgery, the need to control confounders in examining treatment effects (surgery type; here) in data analysis stage; when they cannot be controlled in the study design phase, the use of IPTW which is a suitable method for controlling confounders in data analysis and has received less attention from researchers and finally comparing classic and modern modeling methods in propensity score analysis are some of the reasons that confirm the necessity of conducting this research and its innovativeness.

## Methods

### Design, sample, variables, and data

The present study was a cohort study on colorectal cancer patients. The patients were diagnosed with colon and/or rectal cancer between the years 2011 to the first three months of 2023 and underwent surgery in one of the following hospitals: Shahid Faghihi, Madar and Kodak, Ghadir, and Abu Ali Sina. The inclusion criteria for patients were a definitive diagnosis of colon or rectal cancer, surgery performed using either laparoscopic or laparotomy method, and follow-up visits at least one month after the surgery. The exclusion criteria included the presence of other cancers or inflammatory bowel diseases, and anatomical and structural differences at the surgical site. The final sample size that entered the analysis phase consisted of 916 patients. Considering that the mentioned medical centers for treatment of colorectal cancer patients in the city of Shiraz are actually the references for providing the stated services not only for Fars province population, but also for the south of Iran, it can be said that the results obtained from this sample have acceptable generalizability.

The required information regarding treatment outcomes, type of therapeutic intervention, demographic characteristics, and clinical factors related to cancer was extracted from the Colorectal Cancer Research Center of Shiraz University of Medical Sciences. These variables included cancer region (colon/ rectum), gender (male/ female), cancer history in family, addiction, hypertension, hyperthyroidism, diabetes, hyperlipidemia, asthma, anemia, abdominal pain, constipation, weight loss, rectal bleeding, radiotherapy, chemotherapy, tumor location (right colon, left colon, rectum, total colon), tumor obstruction, tumor differentiation (poor, moderate, well), tumor stage (1/2/3/4), age (years), tumor size (centimeter), involved lymph nodes (number), hemoglobin (grams per deciliter), disease recurrence, metastasis, and death due to cancer. The research protocol of the current study was approved by the Ethics Committee of Shiraz University of Medical Sciences with the code of IR.SUMS.REC.1402.001. Informed consent was obtained from all patients.

### Statistical analysis

In this study, patients with colon and rectal cancer who underwent laparoscopic or open surgeries were compared. To control confounding factors and reduce errors in the study, we used the IPTW analysis as one of the analytical approaches based on PS analysis.

The statistical analysis was conducted in several steps, as follows:

#### 1-Balance analysis prior to IPTW

In the first step, the balance of covariates before weighting was examined. In this stage, confounding variables that had an impact on both treatment allocation and treatment outcomes were analyzed for their balance between the laparoscopic and open surgery using mean difference tests (for continuous variables) and chi-square tests (for categorical variables).

#### 2-Estimation of the propensity scores

In the second step, the PS, which predicts the probability of treatment allocation conditional on covariates, was estimated for each participant, as follows:

$$e\left( x \right) = P\left( {Z = 1|X} \right)$$

When e(x) represents the PS, Z indicates treatment allocation (with a value of 1 indicating treatment allocation (laparoscopic) and 0 indicating non-allocation (open surgery)), and X is a vector of the variables observed. Logistic regression was used to estimate the propensity scores.

$$logit\left( {{Z_i} = 1|X} \right) = {\beta _0} + {\beta _1}{X_{1i}} + \ldots + {\beta _k}{X_{ki}}$$

where the logit represents the logarithm of the odds of receiving treatment (laparoscopic versus open surgery).

$$logit\left({{Z_i} = 1|X} \right) = log (\frac{{P(Zi = 1)}}{{1 - P(Zi = 1)}})$$

The estimated PS, e(x), represents the probabilities of treatment allocation based on the covariates observed, which are derived from the estimated logits.

$${e_i}\left( X \right) = \frac{exp\left(logit\right(Zi=1\left|X\right))}{1+exp\left(logit\right(Zi=1\left|X\right))}$$

Since logistic regression is a predictive model for a binary outcome, the value of e(x) is obtained as a numeric value between 0 and 1.

#### 3-IPTW

In the third step, inverse weighting of the PS was performed. In this method, weights were constructed based on the calculated PSs and assigned to individuals in both treatment groups. The advantage of this method over other approaches is that it utilizes all individuals in both groups; another advantage is that most statistical analysis methods allow for incorporating weights into their calculations [9]. In this approach, individuals in the intervention group (laparoscopic surgery) are assigned a weight of 1/PS, while individuals in the control group (open surgery) are assigned a weight of 1/(1-PS). These weights ensure that for each combination of key characteristics, the sum of the contribution from patients in the intervention and control groups is equal, considering the specific value of PS. For example, consider 20 patients, 6 of whom received the intervention and 14 in the control group. Let’s assume their estimated PS is 0.3. In this case, individuals in the intervention group would have a weight of 1/0.3 = 3.33, and those in the control group would have a weight of 1/0.7 = 1.43. The total weight of the intervention group (6 * 3.33 = 20) equals the total weight of the control group (14 * 1.43 = 20). Therefore, the IPTW creates a synthetic population where the combination of variables in the groups is balanced. This weighting approach helps to reduce the bias caused by confounding variables and ensures comparability between the treatment groups, improving the validity of the analysis.

#### 4-Balance analysis after IPTW

In the fourth step, the balance of variables was re-evaluated after weighting. After applying the IPTW analysis, the distribution of covariates between the treatment groups was assessed to ensure balance.

#### 5-Treatment effects analysis

In the final stage, the effect of laparoscopic surgery compared to laparotomy on postoperative outcomes was estimated using logistic regression in terms of odds ratios.

## Results

From 916 included colorectal cancer patients, open surgery was performed on 216 and laparoscopic surgery on 700 patients. Findings of the differences between laparoscopy and open surgery according to demographic and clinical characteristics before IPTW showed that there were significant differences among the two types of surgery in cancer region, rectal bleeding, tumor location, tumor stage and size (*P* < 0.01), cancer history in family, addiction, hypertension, asthma, abdominal pain, constipation and tumor obstruction (*P* < 0.05), and diabetes and hyperlipidemia (*P* < 0.1) (Table 1). Therefore, covariates the between group imbalances of which was confirmed might be effective on the comparison of postoperative complications such as the status of survival, metastasis, and recurrence of the disease. Thus, treatment effect analysis without controlling this phenomenon leads to confounding bias.

**Table 1:** Balance analysis prior to IPTW analysis

Categorical variables		Frequency (percent)		*P*-value
		Open surgery (*n* = 216)	Laparoscopic surgery (*n* = 700)	
Cancer region	Colon	101 (46.76)	236 (33.71)	0.001^***^
	Rectum	115 (53.24)	464 (66.29)	
Gender	Male	126 (58.33)	408 (58.29)	0.990
	Female	90 (41.67)	292 (41.71)	
Cancer history in family	No	190 (87.96)	572 (81.71)	0.032^**^
	Yes	26 (12.04)	128 (18.29)	
Addiction	No	141 (65.28)	513 (73.29)	0.023^**^
	Yes	75 (34.72)	187 (26.71)	
Hypertension	No	189 (87.50)	564 (80.57)	0.020^**^
	Yes	27 (12.50)	136 (19.43)	
hyperthyroidism	No	208 (96.30)	657 (93.86)	0.172
	Yes	8 (3.70)	43 (6.14)	
Diabetes	No	197 (91.20)	603 (86.14)	0.051^*^
	Yes	19 (8.80)	97 (13.86)	
hyperlipidemia	No	209 (96.76)	653 (93.29)	0.058^*^
	Yes	7 (3.24)	47 (6.71)	
Asthma	No	210 (97.22)	694 (99.14)	0.030^**^
	Yes	6 (2.78)	6 (0.86)	
Anemia	No	193 (89.35)	616 (88.00)	0.589
	Yes	23 (10.65)	84 (12.00)	
Abdominal pain	No	116 (53.70)	442 (63.14)	0.013^**^
	Yes	100 (46.30)	258 (36.86)	
Constipation	No	115 (53.24)	439 (62.71)	0.013^**^
	Yes	101 (46.76)	261 (37.29)	
Weighting loss	No	130 (60.19)	450 (64.29)	0.274
	Yes	86 (39.81)	250 (35.71)	
Rectal bleeding	No	107 (49.54)	257 (36.71)	0.001^***^
	Yes	109 (50.46)	443 (63.29)	
Radiotherapy	No	125 (57.87)	386 (55.14)	0.480
	Yes	91 (42.13)	314 (44.86)	
Chemotherapy	No	131 (60.65)	402 (57.43)	0.402
	Yes	85 (39.35)	298 (42.57)	
Tumor location	Right colon	50 (23.15)	131 (18.71)	< 0.001^***^
	Left colon	47 (21.76)	106 (15.14)	
	Rectum	112 (51.85)	459 (65.57)	
	Total colon	7 (3.24)	4 (0.57)	
Tumor obstruction	No	197 (91.20)	661 (94.43)	0.089
	Yes	19 (8.80)	39 (5.57)	
Tumor Differentiation	Poor	12 (5.56)	31 (4.43)	0.182
	Moderate	61 (28.24)	160 (22.86)	
	Well	143 (66.20)	509 (72.71)	
Tumor stage	1	41 (18.98)	214 (30.57)	< 0.001^***^
	2	79 (36.57)	207 (29.57)	
	3	69 (31.94)	241 (34.43)	
	4	27 (12.50)	38 (5.43)	
Continues variables		Mean (Standard deviation)		*P*-value
Age (years)		56.94 (13.49)	57.10 (13.23)	0.879
Tumor size (centimeter)		4.87 (2.65)	3.81 (2.12)	< 0.001^***^
Involved lymph nodes (number)		1.37 (3.09)	1.02 (2.67)	0.104
Hemoglobin (grams per deciliter)		11.01 (3.15)	10.76 (4.45)	0.438

To balance check, Chi-Square for categorical and t-test for continuous variables were used

Significant on level of ^*^0.1, ^**^0.05 and ^***^0.01

After IPTW analysis, there were no significant differences between open and laparoscopic surgery in terms of clinical and demographic factors (Table 2). Moreover, according to the over-identification test of covariate balance, the null hypothesis that the IPTW analysis balanced all the covariates cannot be rejected (*P* = 0.702). Therefore, after IPTW analysis, treatment analysis of laparoscopic versus open surgery on postoperative complications can be performed without any concern about confounding bias (the last row in Table 2).

**Table 2:** Balance analysis after IPTW analysis

Categorical variables		Frequency (percent)		*P*-value
		Open surgery	Laparoscopic surgery	
Cancer region	Colon	313.9 (33.56)	338.3 (36.74)	0.451
	Rectum	621.3 (66.44)	582.6 (63.26)	
Gender	Male	563.8 (60.28)	539.1 (58.54)	0.704
	Female	371.5 (39.72)	381.8 (41.46)	
Cancer history in family	No	750 (80.19)	767.9 (83.39)	0.428
	Yes	185.2 (19.81)	153 (16.61)	
Addiction	No	676.5 (72.34)	651.1 (70.7)	0.684
	Yes	258.7 (27.66)	269.8 (29.3)	
Hypertension	No	735.3 (78.62)	758.9 (82.41)	0.367
	Yes	200 (21.38)	162 (17.59)	
hyperthyroidism	No	888.4 (94.99)	870.4 (94.51)	0.838
	Yes	46.88 (5.01)	50.52 (5.49)	
Diabetes	No	819.3 (87.6)	806.3 (87.56)	0.990
	Yes	116 (12.4)	114.6 (12.44)	
hyperlipidemia	No	907.1 (96.99)	868 (94.25)	0.129
	Yes	28.15 (3.01)	52.91 (5.75)	
Asthma	No	912.5 (97.56)	902 (97.95)	0.802
	Yes	22.79 (2.44)	18.89 (2.05)	
Anemia	No	832.6 (89.02)	816.5 (88.66)	0.901
	Yes	102.7 (10.98)	104.4 (11.34)	
Abdominal pain	No	563.2 (60.21)	561.2 (60.94)	0.872
	Yes	372.1 (39.79)	359.7 (39.06)	
Constipation	No	598.2 (63.96)	560.8 (60.9)	0.480
	Yes	337.1 (36.04)	360 (39.1)	
Weighting loss	No	577.9 (61.79)	583 (63.31)	0.740
	Yes	357.3 (38.21)	337.9 (36.69)	
Rectal bleeding	No	357.5 (40.15)	368 (39.97)	0.966
	Yes	559.7 (59.85)	552.9 (60.03)	
Radiotherapy	No	528.9 (56.55)	513.7 (55.79)	0.869
	Yes	406.4 (43.45)	407.2 (44.21)	
Chemotherapy	No	555.3 (59.38)	536.6 (58.27)	0.809
	Yes	379.9 (40.62)	384.3 (41.73)	
Tumor location	Right colon	173.2 (18.52)	181.8 (19.75)	0.858
	Left colon	135.8 (14.52)	151 (16.4)	
	Rectum	614.9 (65.74)	575.4 (62.49)	
	Total colon	11.45 (1.22)	12.62 (1.37)	
Tumor obstruction	No	885.8 (94.71)	863.5 (93.76)	0.585
	Yes	49.44 (5.29)	57.42 (6.24)	
Tumor Differentiation	Poor	48.62 (5.2)	43.28 (4.7)	0.956
	Moderate	230.1 (24.61)	222.3 (24.13)	
	Well	656.5 (70.2)	655.4 (71.17)	
Tumor stage	1	250.8 (26.81)	257.2 (27.93)	0.954
	2	282.9 (30.25)	284 (30.83)	
	3	336.5 (35.98)	310.6 (33.73)	
	4	65.05 (6.96)	69.09 (7.5)	
Continues variables		Mean (Standard deviation)		*P*-value
Age (years)		56.99 (1.04)	57.24 (0.55)	0.834
Tumor size (centimeter)		4.05 (0.17)	4.08 (0.11)	0.893
Involved lymph nodes (number)		1.07 (0.15)	1.08 (0.12)	0.971
Hemoglobin (grams per deciliter)		10.60 (0.40)	10.80 (0.17)	0.653
Over identification test for covariate balance*		**Chi-Square value = 25.451**		**0.702**

To balance check, Chi-Square for categorical and Independent two samples t-test for continuous variables were used

*H0: Covariates are balanced

The differences of postoperative outcomes between open surgery and laparoscopic surgery after IPTW are shown in the Table 3. There were significant differences between the two groups of surgery in the percentages of death due to cancer (*P* < 0.01), recurrence (*P* < 0.01), and metastasis (*P* < 0.05). Negative post-operative outcomes were significantly lower in laparoscopic than open surgery. In other words, although 18.25% of patients under laparoscopic surgery were dead due to cancer, this rate was 35.16% for patients under open surgery. Moreover, recurrence rate was lower in laparoscopic surgery (6.78%) than open surgery (17.47%) patients. As to metastasis, its rate in open surgery and laparoscopic surgery patients was 25.72 and 17.47%, respectively.

**Table 3:** Postoperative outcome differences between open and laparoscopic surgery after IPTW analysis

Postoperative outcomes		Frequency (percent)		*P*-value
		Open surgery	Laparoscopic surgery	
Death due to cancer	No	606.4 (64.84)	752.8 (81.75)	< 0.001^***^
	Yes	328.8 (35.16)	168.1 (18.25)	
Recurrence	No	771.9 (82.53)	858.5 (93.22)	< 0.001^***^
	Yes	163.4 (17.47)	62.43 (6.78)	
Metastasis	No	694.7 (74.28)	760 (82.53)	0.028^**^
	Yes	240.5 (25.72)	160.9 (17.47)	

Significant on level of ^**^0.05 and ^***^0.01

Table 4 displays the treatment effect of laparoscopic versus open surgery in colorectal cancer patients using univariate logistic regression analysis. Findings showed that laparoscopic surgery reduced the risk of negative postoperative outcomes including death due to cancer (OR = 0.411, *P* < 0.01), recurrence (OR = 0.343, *P* < 0.01) and metastasis (OR = 0.611, *P* < 0.05) compared to open surgery.

**Table 4:** Treatment effect analysis of laparoscopic surgery vs. open surgery on the postoperative outcomes using univariate logistic regression after IPTW analysis

Postoperative outcomes	Laparoscopic vs. open surgery		
	Odds ratio	95% CI	*P*-value^a^
Death due to cancer	0.411	0.272 0.622	< 0.001^***^
Recurrence	0.343	0.195 0.603	< 0.001^***^
Metastasis	0.611	0.392 0.951	0.029^**^

^a^ for the treatment effect’s regression coefficient

Significant on level of ^**^0.05 and ^***^0.01

## Discussion

In this study, the postoperative complications including the cancer-related mortality, metastasis and recurrence of the disease in patients with colorectal cancer were compared between laparoscopic and open surgery while controlling for confounding variables using the IPTW analysis. Unlike PS matching, the IPTW method does not reduce the sample size and effectively controls the effects of confounding variables while estimating treatment effects with minimum selection bias.

In the current study, IPTW analysis included 24 clinical and demographic factors consisting of cancer region, gender, cancer history in family, addiction, hypertension, hyperthyroidism, diabetes, hyperlipidemia, asthma, anemia, abdominal pain, constipation, weight loss, rectal bleeding, radiotherapy, chemotherapy, tumor location, tumor obstruction, tumor differentiation, tumor stage, age, tumor size, involved lymph nodes, and hemoglobin.

The findings of treatment effect analysis demonstrated that the mortality rate due to cancer in patients who had undergone laparoscopic surgery was significantly lower compared to open surgery. Various studies with different study designs have reported conflicting findings in this regard. Some of these studies were RCT, some were meta-analyses, and a few of them utilized PS analysis methods. Many studies have reported no significant difference in mortality between the two surgical methods. For example, in an RCT conducted in 29 European hospitals, it was shown that there was no significant difference in disease-free survival at 3 years [10]. Another meta-analysis did not report a significant difference in overall mortality or cancer-related mortality [11]. In an international trial conducted in 30 hospitals, it was demonstrated that the two methods were similar in terms of disease-free and overall survival [12]. A prospective randomized trial did not find a significant difference in morbidity or mortality between the two groups [13]. In a PS-matched case-control study on patients aged 80 and above in 41 hospitals, disease-free survival and cancer-specific survival were estimated to be similar between the two groups [14]. Chern et al. (2022), using competing risk analysis in a weighted PS cohort study, showed no significant difference in overall survival and disease-free survival between the two groups in elderly individuals. On the other hand, some studies have shown a clear advantage of the laparoscopic method over open surgery in terms of mortality outcomes, which is consistent with the present study [15]. Kautman et al. (2022), through weighted analysis, demonstrated that laparoscopic methods were associated with a reduced risk of mortality within 90 days compared to open surgery. This advantage in terms of long-term mortality and morbidity persisted for the laparoscopic approach [16]. In a meta-analysis on 15 RCTs, it was shown that 30-day mortality was significantly lower in the laparoscopic group compared to open surgery [17]. In another meta-analysis focusing on elderly patients, three-year survival was found to be higher in the laparoscopic group compared to open surgery [18].

Furthermore, the findings indicated that the rate of recurrence and metastasis in patients who had undergone laparoscopic surgery was significantly lower than in open surgery. There have been few studies conducted on these outcomes, and the majority of studies have shown no significant difference between the two methods. In a meta-analysis of 14 RCTs, it was shown that there was no significant difference between the two surgical groups in terms of the region, port site or wound metastasis, and distant metastasis (*P* = 0.08). However, the recurrence rate was lower in the laparoscopic group (at 10% significance level) [19]. In an international trial, no significant difference was found between the two surgeries in terms of the rates of locoregional recurrence [12]. In a retrospective comparative study, there was no significant difference between the two groups in terms of local recurrence [20]. In an older meta-analysis, no significant difference was found in the disease recurrence rate between the two surgeries [6]. In a prospective nonrandomized trial on patients with extraperitoneal rectal cancer, it was shown that the local recurrence rate was significantly lower in the laparoscopic method (3.2% versus 12.6%) [21].

In more recent studies, although there are contradictions, the findings are more in favor of the laparoscopic surgery rather than open surgery.

Suda et al. (2022) conducted a study on 759 patients with stage 1 to 3 and compared the effectiveness of open surgery with laparoscopic surgery using PS analysis. The number of patients remaining in the study after matching was 460. The results showed that in laparoscopic surgery, intraoperative blood loss, postoperative small bowel obstruction, and postoperative hospitalization are reduced compared to open surgery. In terms of other indicators, there was no significant difference [22]. In a study, video laparoscopic surgery was compared to open surgery in obese patients with colorectal cancer after PS matching. Lower overall mortality was reported in the laparoscopic method [23]. In a study on patients in the first stages of the colorectal cancer, the laparoscopic surgery was associated with lower morbidity and mortality rates [24]. A recently conducted systematic review with 24 studies on elderly colorectal cancer patients, the outcomes of treatment in laparoscopic surgery were better than open surgery, so that the risk of death (RR = 0.7) and the pooled risk of complications (RR = 0.66) were significantly reported lower in these patients [25]. In another new study on 107 totally laparoscopic ileostomy reversal patients compared to open surgery, it showed satisfactory results in terms of short-term outcomes [26]. In a prospective cohort of 137 patients in Sri Lanka, it was shown that although there was no difference in 3-year survival rates, in terms of other indicators laparoscopic surgery is an acceptable method in the treatment of colorectal cancer patients [27]. In a prospective cohort in America on elderly people over 80 years old with colorectal cancer, although there was no difference in 1-month and 12-month mortality, laparoscopic surgery was associated with lower length of stay and hospitalization costs than open surgery [28]. In a new 2023 meta-analysis of 6 studies, they showed that long-term quality of life was not different between the two groups of patients [29].

The conclusion of the literature is that laparoscopic surgery is definitely better in short-term outcomes. However, in long-term outcomes, some studies do not show any differences and some, especially recent researches, are in favor of the laparoscopic treatment.

### Limitations and strengths

In this study, due to the lack of access to data, we were unable to examine further outcomes such as survival analysis. Additionally, the elimination of missing values led to a decrease in sample size. However, one strength of the current study is that we controlled for the effects of 24 clinical and demographic variables, which provides a reasonable level of confidence in the estimated treatment effects.

## Conclusion

The findings of the current study indicate that, in terms of surgical outcomes including cancer-related mortality, recurrence, and metastasis, the laparoscopic surgery outperformed open surgery. Therefore, further development of laparoscopic surgery can lead to better health outcomes for the population and optimize the utilization of healthcare resources. However, the long-term outcomes of laparoscopic versus open surgery should be further considered in the future studies.

## Data Availability

The datasets gathered and analyzed during the current study are available from the corresponding author on reasonable request.

## Abbreviations

GBD: Global Burden of Disease
IPTW: Inverse Probability of Treatment Weighting
PS: Propensity Score
RCT: Randomized Controlled Trial
WHO: World Health Organization
